# Racial and ethnic differences in valuation of life expectancy in prostate cancer treatment decision making

**DOI:** 10.1038/s41391-025-01036-w

**Published:** 2025-10-07

**Authors:** John M. Masterson, Renning Zheng, Michael Luu, Adam Murphy, Yaw A. Nyame, Chad Ritch, Rebecca Gale, Brennan Spiegel, Stephen J. Freedland, Timothy J. Daskivich

**Affiliations:** 1 Department of Urology, Cedars-Sinai Medical Center, Los Angeles, CA USA; 2 Tsinghua Medicine, Tsinghua University, Beijing, China; 3 Department of Biostatistics, Cedars-Sinai Medical Center, Los Angeles, CA USA; 4 Department of Urology, Northwestern University, Chicago, IL USA; 5 Department of Urology, University of Washington, Seattle, WA USA; 6 Division of Public Health Sciences, Fred Hutchinson Cancer Center, Seattle, WA USA; 7 Department of Urology, University of Miami, Miami, FL USA

**Keywords:** Prostate cancer, Outcomes research, Cancer therapy

## Abstract

**Background:**

Life expectancy (LE) is essential for triage between aggressive and conservative management for all prostate cancer risk subtypes. We sought to investigate differences in how Black and Hispanic men interpret LE in treatment decision-making.

**Methods:**

We used targeted crowdsourcing to sample a cohort reflecting sociodemographics of a US prostate cancer population. Subjects completed a conjoint analysis exercise where they iteratively chose between aggressive treatment versus conservative management across levels of 4 tradeoffs—tumor risk (lives saved by aggressive treatment at 5/10/20 year); erectile dysfunction; urinary incontinence; and irritative urinary symptoms—while considering their LE as calculated by the Prostate Cancer Comorbidity Index. Multinomial conditional logistic regression compared odds of choosing aggressive vs. conservative treatment across LEs ranging from 0 to 20 years overall and across racial/ethnic subgroups.

**Results:**

Of 2046 men, 435 (22%) were Black and 230 (11%) were Hispanic. Across all men, the odds of aggressive treatment choice increased by 17% for every 5 years of additional LE (OR = 1.17, 95%CI = 1.12–1.22, *p* < 0.001). Men were significantly more likely to choose aggressive treatment at LE > 13 y and non-aggressive treatment at LE ≤ 10 y. Among Black men, LE was not associated with treatment choice, as they consistently preferred aggressive treatment across all LE categories. Among Hispanic men, increased LE was associated with a higher likelihood of choosing aggressive treatment, with significant preference for aggressive treatment observed only when LE > 10 years. These patterns remained consistent when further stratified by tumor risk.

**Conclusions:**

LE had no impact on treatment decisions in Black men, in contrast to other races and ethnicities. Future research is needed to identify reasons for this phenomenon and to inform culturally relevant approaches to communicating competing mortality risks.

## Introduction

Treatment decision making for localized prostate cancer in men with limited life expectancy (LE) is a clinical dilemma for patients and doctors. Early stage prostate cancer is often slow-growing, diagnosed in older men, and unlikely to represent a mortality risk within the lifetime of the majority of men whose LE is less than 5–10 years [1]. AUA guidelines recommend watchful waiting for asymptomatic men with “limited LE” (i.e. <10 years) but support surgery or radiation for men with intermediate/high-risk disease and LE beyond 10 years [2]. NCCN guidelines integrate LE into treatment recommendations by tumor risk, suggesting observation for men with LE < 10 years and very low to intermediate disease, and for men with LE < 5 years and high to very high-risk disease. Despite these recommendations, it is difficult for men with limited LE and their physicians to weigh the risk of death from cancer against the risk of dying of other causes with potential side effects of treatment. It is not surprising that men with limited LE are both overtreated for lower risk disease and undertreated for higher risk prostate cancer [3, 4].

Previous work has shown that both Black and Hispanic men are less likely to choose active surveillance or watchful waiting and more likely to choose aggressive local therapy compared to non-Hispanic White men for low-risk disease [5]. While these treatment disparities may be partially attributable to provider-level factors, such as lack of cultural competency in communicating LE, differences may also exist at the patient level. Previous studies have found that Black and Hispanic men may have different interpretations and preferences driving these decisions, including how they interpret and integrate LE into decision making [6], which may stem from underlying cultural and experiential factors such as mistrust/distrust in the healthcare system and differences in health literacy or numeracy [7, 8]. However, there is a lack of quantitative analysis isolating the impact of patient race and ethnicity on treatment decision-making, as physician- and patient-level factors are often confounded in real-world clinical consultations.

In this study, we sought to investigate the impact of LE on prostate cancer patient decision making using crowdsourcing of conjoint analysis and then specifically investigated how the impact of LE differed by the race/ethnicity of respondents. We created a conjoint analysis exercise by asking over 2000 men in the public who demographically match a SEER prostate cancer patient population to iteratively choose between aggressive and non-aggressive prostate cancer treatment across varying levels of 4 tradeoffs: tumor risk; erectile dysfunction; urinary incontinence; and irritative urinary symptoms. Subjects were also asked to consider their LE when making these decisions, which was calculated using the Prostate Cancer Comorbidity Index (PCCI), a validated scale for predicting other-cause mortality. We hypothesized that Black and Hispanic men may interpret and integrate LE information differently compared to White populations, reflecting differences in sociocultural factors promoting heightened concern about cancer risk, historical undertreatment of minority populations, and other social differences in lived experiences. If confirmed, this may be a patient-, social-, and healthcare-level factor that touches many issues around race, medicine, and society at large.

## Methods

### Conjoint analysis

**Fig. 1:**
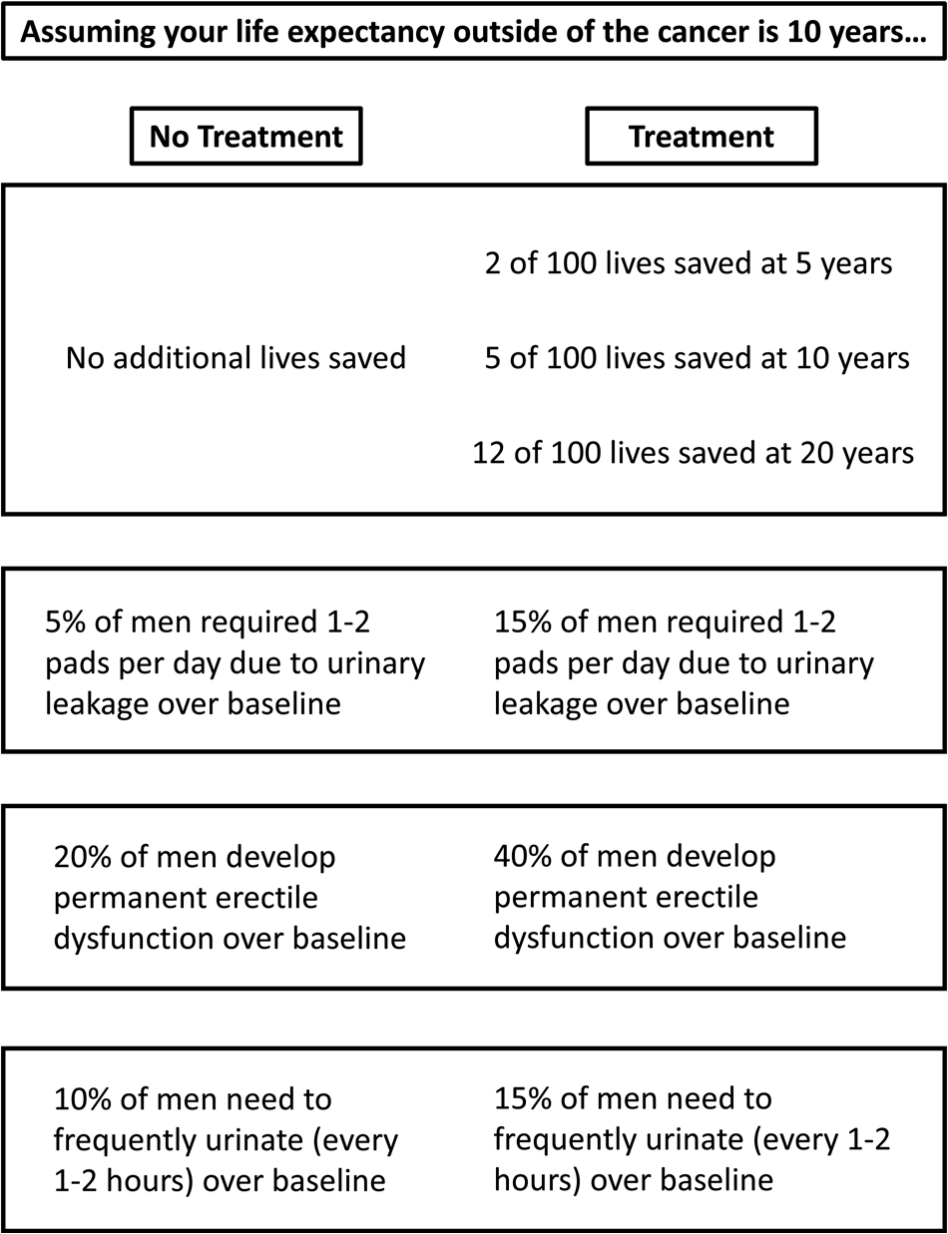
Excerpt from the conjoint analysis exercise administered to our study population.

We used an adaptive choice-based conjoint analysis platform (Sawtooth, North Orem, Utah) to create a conjoint analysis tool for clinically localized prostate cancer. A conjoint analysis is a form of tradeoff analysis that was originally designed for market analysis in how consumers make complex purchasing decisions. This technique has been adapted by healthcare researchers to provide insights into how patients value different decision attributes and has even been integrated into clinical pathways to improve shared decision making [9–11]. Our conjoint model consisted of a computer-based exercise requiring participants to engage in 12 binary choice tasks, each with one option representing “aggressive” treatment (lower risk of 5-,10-,20-year cancer mortality but treatment-related side effects) and the other option representing “non-aggressive” treatment (i.e. no reduction in risk of cancer mortality but no side effects) (Fig. 1).

### Conjoint attributes and levels

The key attributes that were defined for each “aggressive” and “non-aggressive” choice in our models included: (1) tumor risk, expressed as the number of additional lives saved by aggressive treatment out of 100 men at 5, 10, and 20 years (range: 0/0/5, 1/3/9, 2/5/12, 4/8/16, and 5/11/18), (2) risk of urinary incontinence (range: 5%, 10%, 15%, 20%, 25%, 30%, 35%), (3) risk of erectile dysfunction (range: 5%, 20%, 40%, 60%, 80%, 100%), and (4) risk of irritative urinary symptoms (range 5%, 10%, 15%, 20%, 25%, 30%, 35%, 40%). The plausible range of levels for these attributes was defined from outcomes of randomized controlled trials comparing watchful waiting with definitive local therapy and large prospective case series [12–15]. A final attribute that remained fixed for each individual was their projected LE estimated by PCCI [16].

### Covariates

Covariates included age, race/ethnicity, region, household income, education level, health literacy, marital status, medical comorbidity items included in the PCCI, which was used to calculate LE. Health literacy was assessed by the single-item literacy screener (SILS) [17].

### Participants and online crowdsourcing

We partnered with Cint®, a survey research firm with access to multiple research survey panels across the US comprising over 1 million individuals, to capture 2,000 individuals from the community to complete the conjoint analysis exercise. Participants received voucher-based incentives provided by Cint, and no direct incentives was offered by the research team. Requested population characteristics were determined based on the demographic characteristics of a typical US prostate cancer population, including age, race/ethnicity, and regional distribution, according to SEER data [18]. The requested race/ethnicity included 1480 (74%) White, 440 (22%) Black, 60 (3%) Asian/Pacific Islander, and 20 (1%) American Indian/Alaska Native men of whom 1680 (84%) identified as non-Hispanic and 320 (16%) identified as Hispanic. Age distribution included 8 men (0.4%) across all 2000 between ages 35–44, 162 men (8.1%) across all 2000 between ages 45–54, 648 men (32.4%) across all 2000 between ages 55–64, 798 men (39.9%) across all 2000 between 65 and 74, 300 men (15%) across all 2000 between 75 and 84, and 82 men (4.1%) across all 2000 over 84 years.

### Statistical analysis

Baseline patient demographics were described as frequency counts and percentages. Discrete choice modeling was used to analyze the preferences of patients for aggressive treatment versus conservative management across varying levels of 4 tradeoffs: tumor risk (lives saved by aggressive treatment at 5, 10, and 20 years); erectile dysfunction; urinary incontinence; and irritative urinary symptoms. Multinomial conditional logit modeling was used to model patient choice, where the dependent variable was choice among the 12 binary choice tasks for a given patient. Independent variables in the model were attributes as described above adjusting for race, ethnicity, education, income, marital status, health literacy, and age. We separately fit the model including various 2- and 3-way interaction terms including LE*treatment, LE*treatment*race, LE*treatment*ethnicity, tumor risk*LE*race, and tumor risk*LE*ethnicity, to explore the impact of LE on treatment choice separately by race and ethnicity. Using the multinomial conditional logit model, we estimated the marginal mean effects of the impact of LE on treatment choice within race, ethnicity, and tumor risk strata.

Statistical analyses were performed using R software package version 4.2.3. Two-sided *P* values < 0.05 were considered significant.

## Results

In total, 2046 men were included in our analytic sample (Table 1; Supplementary Table 1). 435 (22%) were Black, 230 (11%) were Hispanic. Black participants were younger, single, had less years of education, lower income, and lower comorbidity scores than non-Black participants (Table 1). Hispanic participants were younger, more often married or divorced/separated and less often single, and had lower comorbidity scores than non-Hispanic participants (Supplementary Table 1). 1212 (59%) and 594 (29%) men described themselves as extremely or quite health literate by the SILS, respectively, and there were no differences in healthy literacy by race or ethnicity.

**Table 1:** Sample characteristics by race.

	Overall	Black	White or Other	*P*
	*N* = *2046*	*N* = *435*	*N* = *1446*	
Age				<0.001
35–44	35 (1.72%)	4 (0.92%)	31 (1.94%)	
45–54	169 (8.30%)	71 (16.3%)	98 (6.12%)	
55–64	675 (33.1%)	223 (51.3%)	452 (28.2%)	
65–74	812 (39.9%)	116 (26.7%)	696 (43.4%)	
75–84	287 (14.1%)	20 (4.60%)	267 (16.7%)	
85+	59 (2.90%)	1 (0.23%)	58 (3.62%)	
Median (Q1, Q3)	66 (58, 72)	61 (56, 66)	68 (60, 73)	
Health Literacy				0.3
Extremely	1212 (59.2%)	272 (62.5%)	940 (58.3%)	
Quite a bit	594 (29.0%)	110 (25.3%)	484 (30.0%)	
Somewhat	188 (9.19%)	44 (10.1%)	144 (8.94%)	
A little bit	32 (1.56%)	6 (1.38%)	26 (1.61%)	
Not at all	20 (0.98%)	3 (0.69%)	17 (1.06%)	
Ethnicity				<0.001
Non Hispanic	1812 (88.7%)	426 (97.9%)	1386 (86.2%)	
Hispanic	230 (11.3%)	9 (2.07%)	221 (13.8%)	
Education				<0.001
High School or Lower	940 (45.9%)	243 (55.9%)	697 (43.3%)	
Associate/Bachelors	776 (37.9%)	152 (34.9%)	624 (38.7%)	
Masters/Professional/Doctorate	330 (16.1%)	40 (9.20%)	290 (18.0%)	
Income				<0.001
Less than $25,000	371 (18.1%)	124 (28.5%)	247 (15.3%)	
$25,000 to $49,999	546 (26.7%)	112 (25.7%)	434 (26.9%)	
$50,000 to $74,999	424 (20.7%)	96 (22.1%)	328 (20.4%)	
$75,000 to $99,999	272 (13.3%)	38 (8.74%)	234 (14.5%)	
$100,000 to $149,999	236 (11.5%)	31 (7.13%)	205 (12.7%)	
$150,000 or more	158 (7.72%)	24 (5.52%)	134 (8.32%)	
Prefer not to answer	39 (1.91%)	10 (2.30%)	29 (1.80%)	
Marital status				<0.001
Single, never married	363 (17.7%)	129 (29.7%)	234 (14.5%)	
Married or domestic partnership	1185 (57.9%)	187 (43.0%)	998 (61.9%)	
Widowed	133 (6.50%)	20 (4.60%)	113 (7.01%)	
Divorced/Separated	364 (17.8%)	99 (22.8%)	265 (16.4%)	
Prefer not to answer	1 (0.05%)	0 (0.00%)	1 (0.06%)	
PCCI				<0.001
0	617 (30.2%)	215 (49.4%)	402 (25.0%)	
1	468 (22.9%)	84 (19.3%)	384 (23.8%)	
2	430 (21.0%)	76 (17.5%)	354 (22.0%)	
3	219 (10.7%)	22 (5.06%)	197 (12.2%)	
4	139 (6.79%)	15 (3.45%)	124 (7.70%)	
5+	173 (8.46%)	23 (5.29%)	150 (9.31%)	
Life Expectancy	18.0 [15.0;20.0]	18.0 [16.0;20.0]	16.0 [15.0;18.0]	<0.001

**Fig. 2:**
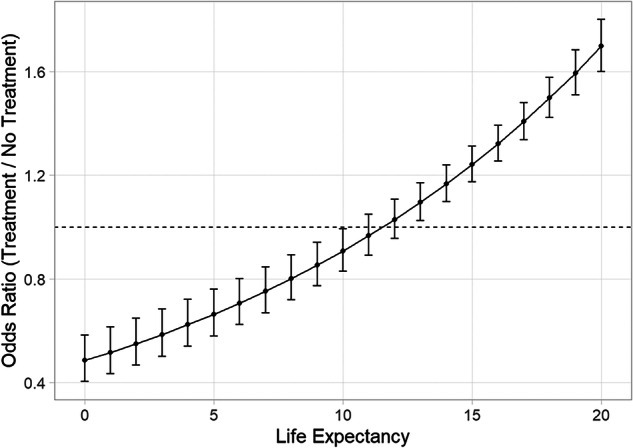
Odds ratio of treatment versus no treatment for clinically localized prostate cancer by patient life expectancy.

Across all men, participants were more likely to opt for prostate cancer treatment as LE increased—the odds of aggressive treatment choice increased by 17% for every 5 years of additional LE (OR = 1.17, 95%CI = 1.12–1.22, *p* < 0.001). Men were significantly more likely to choose aggressive treatment at LE > 13 years and non-aggressive treatment at LE ≤ 10 years (Fig. 2). All tested interaction terms (including LE*treatment, LE*treatment*race, LE*treatment*ethnicity, tumor risk*LE*race, and tumor risk*LE*ethnicity) were statistically significant (all, *p* < 0.001), suggesting that impact of LE on treatment choice may differ by race, ethnicity, and tumor risk.

**Fig. 3:**
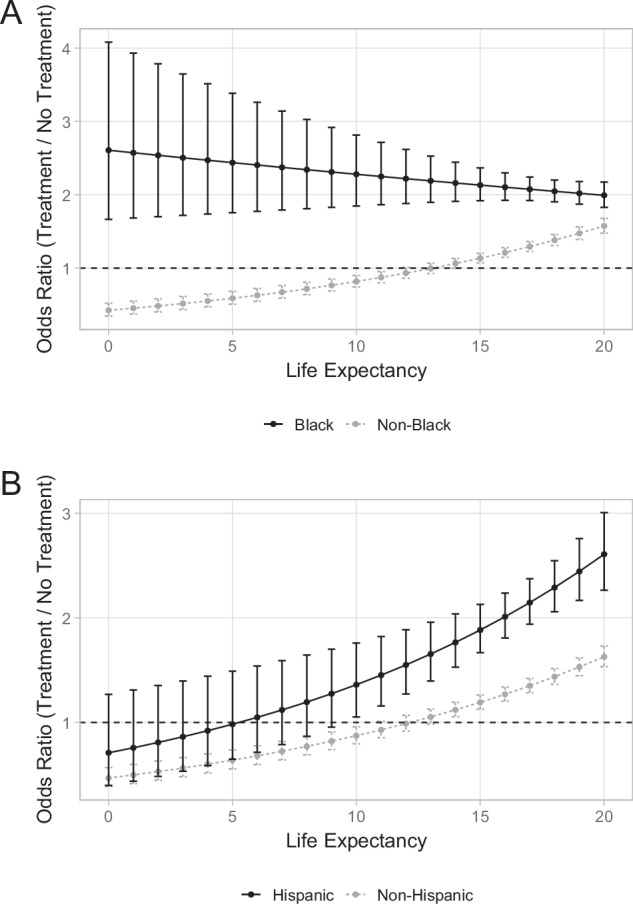
Odds ratio for aggressive treatment choice by life expectancy across race and ethnicity. **A** Odds ratio for Black versus non-Black men; **B** Odds ratio for Hispanic versus non-Hispanic men.

When stratified by race, LE was not associated with treatment choice among Black men (OR = 0.97 for each five additional years of LE, 95%CI = 0.90–1.05, *p* = 0.48), as they consistently preferred aggressive treatment across all LE categories (Fig. 3A). When stratified by ethnicity, increased LE was associated with a higher likelihood of choosing aggressive treatment among Hispanic men (OR = 1.14 for each five additional years of LE, 95%CI = 1.02–1.27, *p* = 0.019), with significant preference for aggressive treatment observed only when LE > 10 years (Fig. 3B).

**Fig. 4:**
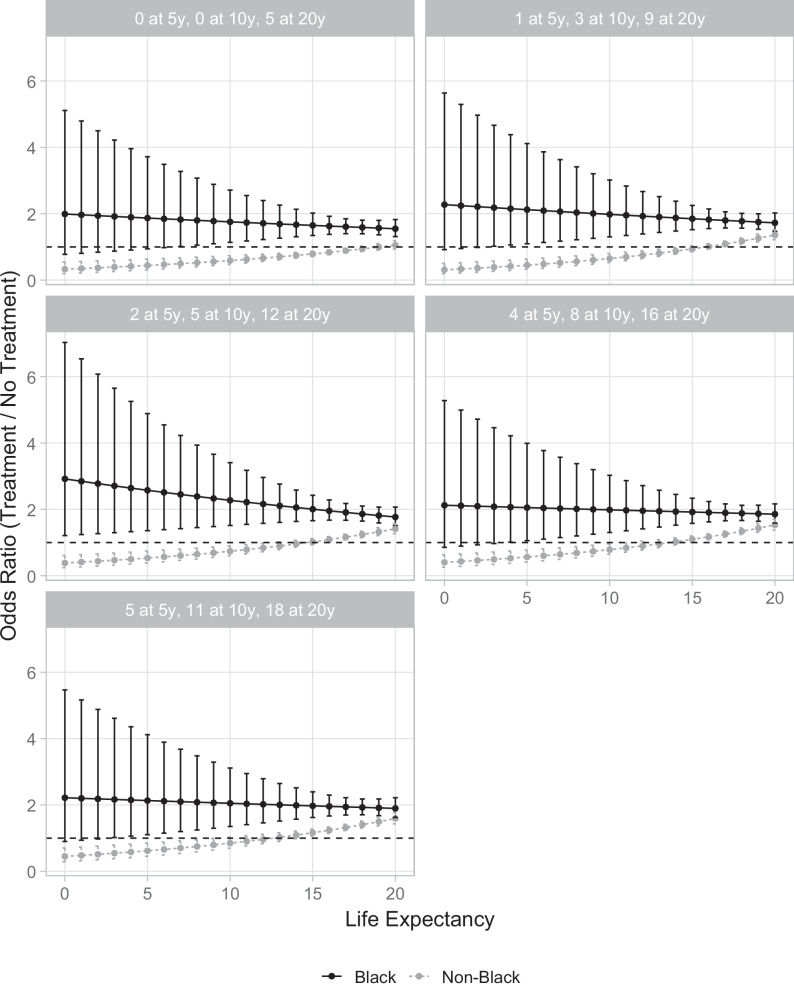
Odds ratio for aggressive treatment choice by life expectancy for Black men versus non-Black men stratified by tumor risk. Tumor risks were represented as the number of additional lives saved by aggressive treatment out of 100 men at 5, 10, and 20 years. Each panel represents increasing tumor risk from (left-to-right, top-to-bottom).

These patterns remained consistent when further stratified by tumor risk. Among Black men, LE remained unassociated with treatment preferences (all, *p* ≥ 0.30) and aggressive treatment was consistently preferred regardless of LE across all tumor risk subgroups (Fig. 4). Similarly, the association between longer LE and the preference for aggressive treatment among Hispanic men persisted when stratified by tumor risk, although the study was underpowered to detect statistically significant differences within tumor risk subgroups (Supplementary Fig. 1).

## Discussion

LE is a critical decision factor for patients and physicians to consider when making decisions regarding treatment of clinically localized prostate cancer, since it is the primary indicator of whether a patient will live long enough to derive significant benefit from aggressive local therapy and has been shown to predict treatment benefit. Previous studies have shown that Black and Hispanic men are often overtreated for low-risk cancer, but the contribution of patient-level drivers of this trend are not well defined. To better understand how Black and Hispanic men interpret and integrate LE when considering aggressive versus conservative management, we crowdsourced a conjoint analysis exercise to a population of men who demographically represent a typical US prostate cancer population based on SEER. Among Black men, LE had no impact on treatment choices, which contrasted with trends observed in White and Hispanic men. Ours is the first such study to show that Black men may interpret and integrate LE differently than other groups when making treatment decisions and may provide insight into this multi-level issue that may in part drive overtreatment for lower risk disease in these men.

Previous studies have explored racial and ethnic differences in prostate cancer management and found that Black men are managed conservatively less often than non-Black men for low risk prostate cancer [5, 19]. Several large SEER studies have shown that Black men with low risk prostate cancer are less likely to be managed with active surveillance or watchful waiting than non-Black men [5, 19, 20]. These differences may be driven by both provider and patient factors. From a provider perspective, possible drivers of this phenomenon may include implicit or explicit racial bias, as well as concerns about higher rates of upgrading/upstaging in Black men [21], increased overall risk of prostate cancer mortality [22], and underrepresentation of Black men in active surveillance study cohorts [23], and lack of cultural competency in communicating competing risks [24, 25]. What has not been previously demonstrated is the degree to which patient preference may drive decisions against conservative management among these men, as prior studies have typically conflated patient-level factors with physician-level influences. Importantly, our study addresses this limitation by performing a conjoint analysis in which all participants received standardized, accurate information about life expectancy, cancer prognosis, and potential side effects. By controlling for physician-level variability, our study isolates the influence of patient preferences on decision-making.

Our data suggest that Black men may be less likely to choose conservative management of their low-risk prostate cancer even when their LE is limited, and they are unlikely to benefit from aggressive treatment. There are likely multiple explanations for why this is the case, which we were unable to capture in our data. One possibility is that Black men may be less likely to trust data favoring non-aggressive treatment of cancer than other groups, either due to general mistrust of the healthcare system or due to awareness of inappropriate undertreatment of cancer in Black populations in other contexts. For example, these men may be skeptical of triage of treatment of low-risk prostate cancer based on LE because they are aware of longstanding undertreatment of higher risk disease in Black men. This perception may be informed by the higher prevalence and risk of prostate cancer in Black men, which makes it more likely for a Black man with low-risk cancer to be influenced by negative outcomes of a family member or friend with higher risk cancer.

Another important patient-level factor that may influence treatment preference is cancer fatalism, a belief commonly observed among Black patients [26] that cancer development is predetermined and that death is inevitable following a cancer diagnosis [27]. However, our findings do not support the idea that cancer fatalism influences treatment choice in this context, as Black patients were more likely to opt for aggressive treatment regardless of their LE. Notably, cancer fatalism encompasses two distinct dimensions: the belief in the inevitability of a cancer diagnosis (occurrence fatalism), and the belief that death is inevitable once cancer is diagnosed (outcome fatalism). These two concepts are often use interchangeably and most studies examining cultural and ethnic differences in fatalism do not distinguish between them [28, 29]. However, previous studies have found patients who believed occurrence fatalism did not necessarily endorsed outcome fatalism [30, 31]. Furthermore, while many previous studies have shown that occurrence fatalism served as a barrier to cancer screening, there is limited research on the effect of outcome fatalism [32]. As such, although our findings do not support an association between outcome fatalism and treatment decision-making among Black patients, whether this is due to a low prevalence of outcome fatalism or to its limited influence on treatment choices warrants further investigation.

Our study has several limitations which should be addressed. First, none of the participants in our study have a diagnosis of prostate cancer but are members of the public. While individuals without cancer in the community may interpret and integrate decision attributes differently than those with cancer, there is important precedent for using the general public to assign value to health states [33]; in the United Kingdom, crowdsourcing was used in the development of the EuroQol-5D (EQ-5D) to obtain utility values for health states, which informs decisions regarding public payer coverage through the National Institute for Health and Clinical Excellence (NICE) [34]. Furthermore, there is a robust experience in the use of conjoint analysis assessing patient preferences in numerous disease states, including prostate cancer [35]. Second, given the overall high level of healthy literacy among the population, participants may have some prior knowledge of prostate cancer natural history through family members, general practitioners, or the media – which may affect their decisions within the conjoint analysis exercise. Third, although our conjoint analysis accounted for key clinical factors for treatment decision-making including LE, tumor risk, and side effects, some other potentially influential variables such as past medical history were not captured. Fourth, race and ethnicity are imperfect proxies for individual cultural and experiential factors that may contribute to differences in treatment preferences. However, we were not able to directly assess the impact of these factors due to the lack of relevant data to measure them. Finally, although we only used a single-item self-reported measure of health literacy, its consistency with objective measures of literacy has been previously validated. While we did not specifically measure numeracy, previous work by our group has shown strong correlation between patient health literacy and numeracy in similar crowdsourced populations [17, 36].

## Conclusions

LE is an essential component of shared decision-making in the management of localized prostate cancer, since it identifies men with limited expected longevity who may not live long enough to benefit from treatment. Our data suggest that Black men interpret and integrate LE differently in the context of prostate cancer decisions, unlike Hispanic and Caucasian populations. This may be driven by sociocultural factors including mistrust/distrust in the health care system, concerns regarding historical undertreatment of minority populations, heightened perceptions of cancer lethality among Black men, and negative experiences of family or friends with prostate cancer. Validated tools to assess these sociocultural factors, as well as qualitative or mixed-methods studies, are essential to contextualize preferences in future research. While our study cannot parse the exact reason for this observation and should be interpreted as exploratory, further research is needed to better understand why Black men would opt for aggressive treatment despite shorter LE and limited treatment benefits. These findings call for development of a culturally relevant approach to risk communication that can improve shared decision-making and guideline concordant care for these men.

## Supplementary information

Supplementary Materials

## Data Availability

The datasets generated during and/or analyzed during the current study are available from the corresponding author on reasonable request.
