# Supplementary material for: Racial and ethnic differences in valuation of life expectancy in prostate cancer treatment decision making
Source: Prostate Cancer Prostatic Dis. 2025 Oct 7;29(2):397–403. doi: 10.1038/s41391-025-01036-w (PMC13190296; doi:10.1038/s41391-025-01036-w)
Supplement: Supplementary file 1 — Supplementary Materials [file 41391_2025_1036_MOESM1_ESM.docx]

**Supplementary Table 1.** Sample characteristics by Ethnicity

|  | **Overall** | **Hispanic** | **Non Hispanic** | **P** |
| --- | --- | --- | --- | --- |
|  | *N=2042* | *N=230* | *N=1812* |  |
| Race |  |  |  | <0.001 |
| Black | 435 (21.3%) | 9 (3.91%) | 426 (23.5%) |  |
| White | 1445 (70.8%) | 162 (70.4%) | 1283 (70.8%) |  |
| Other | 162 (7.93%) | 59 (25.7%) | 103 (5.68%) |  |
| Age |  |  |  | 0.007 |
| 35-44 | 35 (1.72%) | 4 (1.78%) | 31 (1.71%) |  |
| 45-54 | 169 (8.31%) | 21 (9.33%) | 148 (8.19%) |  |
| 55-64 | 673 (33.1%) | 85 (37.8%) | 588 (32.5%) |  |
| 65-74 | 811 (39.9%) | 96 (42.7%) | 715 (39.5%) |  |
| 75-84 | 287 (14.1%) | 19 (8.44%) | 268 (14.8%) |  |
| 85+ | 58 (2.85%) | 0 (0.00%) | 58 (3.21%) |  |
| Median (Q1, Q3) | 66 (58, 72) | 66 (59, 73) | 65 (57, 69) |  |
| Health Literacy |  |  |  | 0.2 |
| Extremely | 1210 (59.3%) | 135 (58.7%) | 1075 (59.3%) |  |
| Quite a bit | 592 (29.0%) | 60 (26.1%) | 532 (29.4%) |  |
| Somewhat | 188 (9.21%) | 24 (10.4%) | 164 (9.05%) |  |
| A little bit | 32 (1.57%) | 6 (2.61%) | 26 (1.43%) |  |
| Not at all | 20 (0.98%) | 5 (2.17%) | 15 (0.83%) |  |
| Education |  |  |  | 0.3 |
| Associate/Bachelors | 774 (37.9%) | 97 (42.2%) | 677 (37.4%) |  |
| Masters/Professional/Doctorate | 329 (16.1%) | 32 (13.9%) | 297 (16.4%) |  |
| Other | 939 (46.0%) | 101 (43.9%) | 838 (46.2%) |  |
| Income |  |  |  | 0.6 |
| Less than $25,000 | 369 (18.1%) | 40 (17.4%) | 329 (18.2%) |  |
| $25,000 to $49,999 | 545 (26.7%) | 63 (27.4%) | 482 (26.6%) |  |
| $50,000 to $74,999 | 424 (20.8%) | 45 (19.6%) | 379 (20.9%) |  |
| $75,000 to $99,999 | 272 (13.3%) | 34 (14.8%) | 238 (13.1%) |  |
| $100,000 to $149,999 | 235 (11.5%) | 30 (13.0%) | 205 (11.3%) |  |
| $150,000 or more | 158 (7.74%) | 17 (7.39%) | 141 (7.78%) |  |
| Prefer not to answer | 39 (1.91%) | 1 (0.43%) | 38 (2.10%) |  |
| Marital Status |  |  |  | 0.003 |
| Single, never married | 362 (17.7%) | 29 (12.6%) | 333 (18.4%) |  |
| Married or domestic partnership | 1183 (57.9%) | 145 (63.0%) | 1038 (57.3%) |  |
| Widowed | 133 (6.51%) | 7 (3.04%) | 126 (6.95%) |  |
| Divorced/Separated | 363 (17.8%) | 48 (20.9%) | 315 (17.4%) |  |
| Prefer not to answer | 1 (0.05%) | 1 (0.43%) | 0 (0.00%) |  |
| PCCI |  |  |  | 0.049 |
| 0 | 616 (30.2%) | 71 (30.9%) | 545 (30.1%) |  |
| 1 | 468 (22.9%) | 70 (30.4%) | 398 (22.0%) |  |
| 2 | 430 (21.1%) | 39 (17.0%) | 391 (21.6%) |  |
| 3 | 218 (10.7%) | 24 (10.4%) | 194 (10.7%) |  |
| 4 | 139 (6.81%) | 11 (4.78%) | 128 (7.06%) |  |
| 5+ | 171 (8.37%) | 15 (6.52%) | 156 (8.61%) |  |
| Life Expectancy | 18.0 [15.0;20.0] | 18.0 [16.0;20.0] | 18.0 [15.0;20.0] | 0.09 |


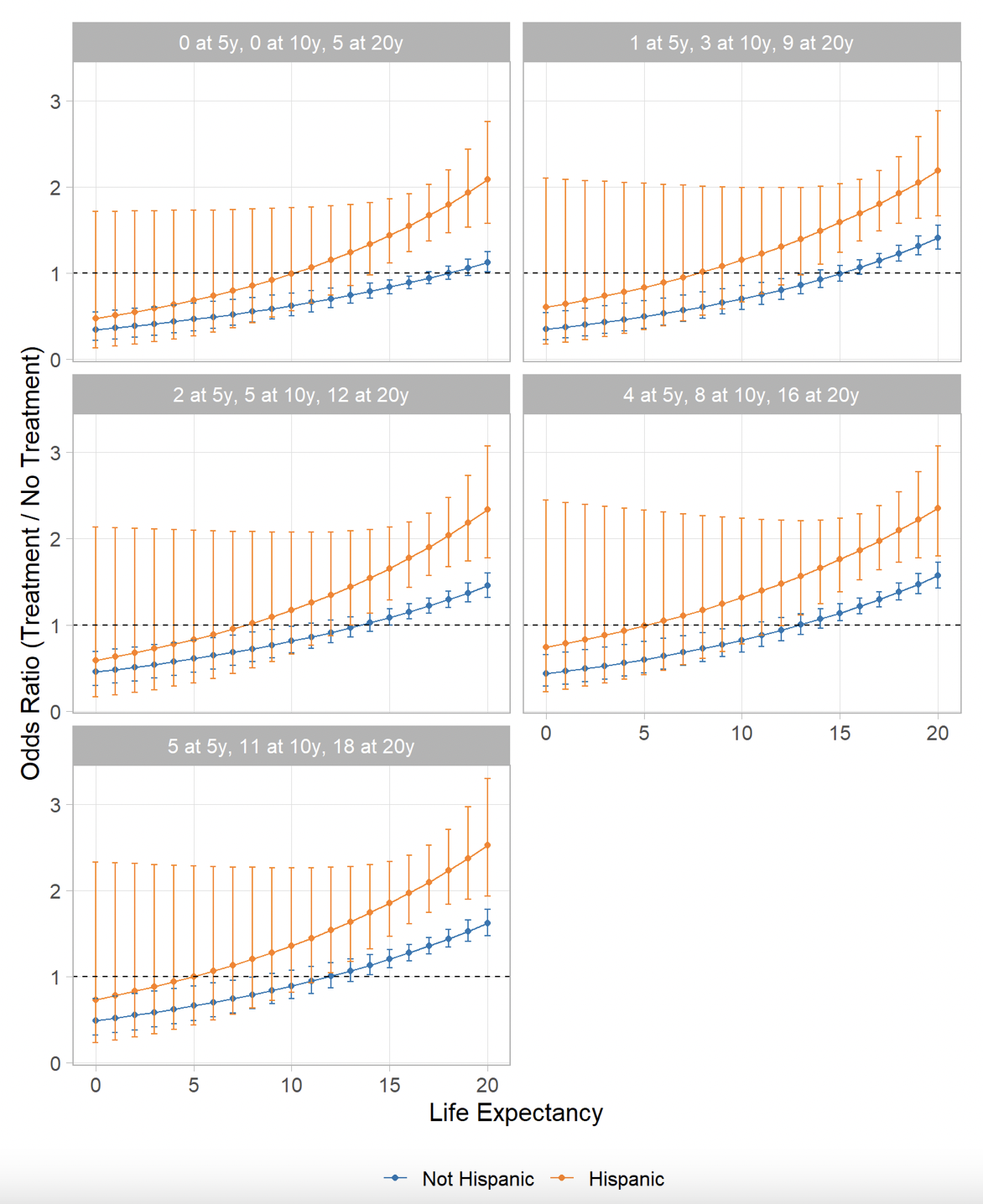


**Supplementary Figure 1.** Odds Ratio for aggressive treatment choice by life expectancy for Hispanic men versus non-Hispanic men

Tumor risks were represented as the number of additional lives saved by aggressive treatment out of 100 men at 5, 10, and 20 years. Each panel represents increasing tumor risk from (left-to-right, top-to-bottom).
